# *Mycoplasma bovis* is associated with *Mannheimia haemolytica* during acute bovine respiratory disease in feedlot cattle

**DOI:** 10.3389/fmicb.2022.946792

**Published:** 2022-08-01

**Authors:** Robert Valeris-Chacin, Sherri Powledge, Taylor McAtee, Paul S. Morley, John Richeson

**Affiliations:** ^1^Veterinary Education, Research, and Outreach (VERO), Department of Veterinary Pathobiology, College of Veterinary Medicine and Biomedical Sciences, Texas A&M University, College Station, TX, United States; ^2^Department of Agricultural Sciences, Paul Engler College of Agriculture and Natural Sciences, West Texas A&M University, Canyon, TX, United States; ^3^Veterinary Education, Research, and Outreach (VERO), Department of Large Animal Clinical Sciences, College of Veterinary Medicine and Biomedical Sciences, Texas A&M University, Canyon, TX, United States

**Keywords:** *Mycoplasma bovis*, acute BRD, beef cattle, IPW models, *Mannheimia haemolytica*

## Abstract

Bovine Respiratory Disease (BRD) represents a significant burden to the health of feedlot cattle and the profitability of the beef industry in the US. *Mannheimia haemolytica* is widely regarded as the primary bacterial pathogen driving acute BRD. While *Mycoplasma bovis* is most commonly implicated in chronic cases of BRD, this agent's potential role in acute stages of BRD is unclear. Therefore, this study aimed to evaluate potential associations between *M. bovis* and *M. haemolytica* during acute BRD in feedlot cattle. Nasal swabs (*n* = 1,044) were collected over time from feedlot cattle (*n* = 270) enrolled in an experiment assessing the effect of vaccination for Bovine Respiratory Syncytial Virus (BRSV). Swabs were analyzed for detection of *M. bovis, M. haemolytica, Pasteurella multocida, Histophilus somni*, and BRSV *via* multiplex qPCR assays. Data were analyzed using inverse conditional probability weighted (ICPW) logistic regression models to investigate potential effects of *M. bovis* presence on arrival (d0), day seven (d7) and day 14 (d14) post-arrival on *M. haemolytica* prevalence on day 28 (d28) post-arrival, adjusting for the previous history of *P. multocida, H. somni*, BRSV, BRD morbidity, and body weight. The potential association between time-to-BRD detection and *M. bovis* presence on d0, d7, and d14 post-arrival, was inferred *via* an ICPW time-to-event model. The presence of *M. bovis* in nasal swabs collected on d7 post-arrival was significantly associated with an increase in the prevalence of *M. haemolytica* on d28 (prevalence difference: 45%; 95% Confidence Interval: 31%, 60%; *P*-value < 0.001). Significant time-varying coefficients for *M. bovis* presence were detected at d0, d7, and d14 post-arrival in the ICPW time-to-event model (*P*-value < 0.001). The shortest median time-to-BRD detection was 29 days in cattle that were *M. bovis* positive on d0, d7, and d14 post-arrival and in those that were positive on d0 and d14 post-arrival. Under the conditions of this study, our findings suggest that *M. bovis* may be influencing the respiratory environment during the acute phase of BRD, increasing the abundance of *M. haemolytica*, which could have important impacts on the occurrence of BRD.

## Introduction

Bovine respiratory disease (BRD) represents an enormous animal welfare issue and an economic burden to beef cattle production in the US (Peel, 2020). It is estimated that BRD morbidity is 16.2% in the feedlots (USDA, 2013). BRD mortality accounts for 55 and 36.3% of non-predator mortality in cattle and calves, respectively, in feedlots and backgrounding operations in the US (USDA, 2017). Additionally, BRD is the disease most frequently associated with antimicrobial drug (AMD) treatment in beef cattle (Brault et al., 2019). Common clinical signs of BRD are nasal discharge, depression, dyspnea, fever, appetite loss, and even acute mortality (Griffin et al., 2010).

Several viruses (BHV-1, BVDV, PI3V, BRSV) and bacteria (*Mannheimia haemolytica, Histophilus somni, Pasteurella multocida*, and *Mycoplasma bovis*) have been associated with BRD (Angen et al., 2009; Gershwin et al., 2015; Cirone et al., 2019). Interestingly, these bacterial agents are commonly present in the upper respiratory tract of healthy animals and could be considered to be commensals until other factors triggers the BRD pathogenesis (Confer, 2009; Bakaletz, 2017; Lion et al., 2021). Among the BRD bacterial agents, *M. haemolytica* is the most relevant in acute BRD, causing fibrinonecrotizing lesions in the lungs (Rice et al., 2007; Booker et al., 2008; Mason et al., 2022).

*Mycoplasma bovis* is a bacterium member of the family Mycoplasmataceae, characterized by a small genome, lack of cell wall, and high nutritional requirements for *in vitro* growth (Li et al., 2011; Parker et al., 2018; Dudek et al., 2020). Traditionally, *M. bovis* has most typically been linked to chronic BRD with characteristic pneumonic lesions that are often non-responsive to AMD therapy (Booker et al., 2008; Hermeyer et al., 2012a; Gershwin et al., 2015; Becker et al., 2020). Several virulence factors have been implicated in the ability of *M. bovis* to persist in the lungs of cattle with BRD, such as variable surface proteins (Vsp), adhesins, nucleases, H_2_O_2_ production, and biofilm formation (Burki et al., 2015; Perez-Casal, 2020). Due to the difficulties of growing *M. bovis* in the lab, the associated culture bias (Prakash et al., 2021) may have falsely shaped our prior understanding of the role of *M. bovis* in BRD pathogenesis.

Despite accepted dogma regarding *M. bovis* as a pathogen associated with chronic pneumonia in cattle, recent evidence has emerged linking *M. bovis* presence in the upper respiratory tract with acute BRD status (Timsit et al., 2018; Centeno-Martinez et al., 2022; Crosby et al., 2022). However, the influence of *M. bovis* on other members of the microbial community of the respiratory tract, especially other BRD agents such as *M. haemolytica*, remains unknown shortly after arrival and mixing with other cattle at feedlots. Therefore, this study evaluated the associations of *M. bovis* and *M. haemolytica* during the early feeding period and acute BRD occurrence in feedlot cattle. A more comprehensive understanding of the dependencies among BRD pathogens over time will help develop new non-antibiotic control strategies and subsequently reduce the burden of BRD on beef cattle production.

## Materials and methods

### Experimental design

Crossbred beef bull and steer calves that were considered to have a high risk for BRD based upon demographics and prior experience were purchased at an auction market and then enrolled in a randomized controlled vaccine efficacy study; these animals were also used to address this study's objectives. For that vaccine trial (Powledge et al., 2022), cattle were randomly assigned to three treatment groups: an unvaccinated negative control group, a group vaccinated intranasally with a trivalent modified-live virus (MLV) respiratory vaccine (BHV-1, BRSV, and PI3V) with parenteral BVDV type I and type II vaccine, and a group vaccinated with a parenteral pentavalent MLV respiratory vaccine (BHV-1, BRSV, PI3V, BVDV type I and type II). Treatment was applied the following morning after arrival (6:00 am CST), but that day will be equated to arrival for simplicity. The random allocation of treatment was stratified by individual body weight (BW), sex (bull or steer), and presence/absence of a pre-existing ranch tag. Additionally, stratification by the truckload (total of five truckloads) was performed. Treatment pen assignment (15 pens per treatment group) was spatially arranged to minimize unwanted MLV transmission between the vaccine treatments and the negative control group. Tildipirosin (Zuprevo, Merck Animal Health, NJ, USA) was administered as BRD metaphylaxis to all cattle as per label.

A total of 12 cattle were randomly assigned to each pen, six of which were also randomly selected for sampling (*n* = 270 cattle selected for sampling). Nasal swabs were collected on days 0, 7, 14, and 28 post-arrival. Nasal swabs (*n* = 1,044 swabs) were collected by personnel blinded to treatment allocation and stored at −20°C until submitted for analysis at the Texas Veterinary Medicine Diagnostic Laboratory (TVMDL) in Canyon, TX. Nasal swabs were used for molecular detection of BRSV, *H. somni, M. haemolytica, P. multocida*, and *M. bovis via* a multiplex qPCR with TaqMan chemistry (Applied Biosystems, MA, USA).

Using a clinical illness score (CIS) (Pillen et al., 2016), personnel blinded to treatment allocation evaluated cattle daily for BRD symptoms. Cattle with a CIS of 2 or 3 were administered florfenicol (40 mg/kg BW, Nuflor, Merck Animal Health). Enrofloxacin (11 mg/kg BW, Baytril, Bayer Animal Health, KS, USA) was administered as a second antimicrobial treatment to those animals with a CIS of 2 or 3 after 3 days of the treatment with florfenicol. Ceftiofur (6.6 mg/kg BW, Excede, Zoetis, NJ, USA) was administered in case a third antimicrobial treatment was needed (CIS of 2 or 3 after 3 days of the treatment with enrofloxacin). Cattle with a CIS of 4 were humanely euthanized. Cattle deemed chronically ill (three antimicrobial treatments combined with <1.0 lb in average daily gain since arrival or body condition scoring <3) were removed from the study.

### Statistical analysis

The molecular detection of BRSV, *H. somni, M. haemolytica, P. multocida*, and *M. bovis* generated continuous variables (Cycle threshold or Ct value) and binary variables (presence/absence) based on a cutoff value (Ct value of 36) validated by the TVMDL. Any instance of no detection, i.e., below the limit of detection of the qPCR assay, was assigned the highest Ct value possible (Ct value of 40).

Mixed-effects logistic regression was used to evaluate the longitudinal associations of anti-BRSV vaccination with the prevalence of each pathogen, calculating the prevalence difference (PD) as a measure of association. Therefore, each pathogen's presence/absence variable was regressed on treatment group, time (sampling day), and their interaction, as fixed effects. Block, pen, and animal were used as nested random intercepts. Similarly, mixed-effects linear regression was used to assess the longitudinal associations of BRSV vaccination with the amount of DNA detected for each pathogen, using the Ct values as a proxy. These mixed-effects linear models used the same variables described for the mixed-effects logistic regression, using restricted maximum likelihood parameter estimation with Kenward-Roger method to calculate degrees of freedom. Additional mixed-effects linear regression models, in which *M. haemolytica* Ct values were regressed on either *M. bovis, H. somni, P. multocida*, or BRSV Ct values, adjusting for treatment and time, were also evaluated, using the model parameters previously described. Likelihood ratio tests were used to select the random effects retained in the final models (Self and Liang, 1987).

The potential effects of the presence of *M. bovis* on *M. haemolytica* prevalence and Ct values on day 28 post-arrival, mortality, and BRD morbidity (on days 14 and 28 post-arrival) were evaluated using inverse conditional probability weighted (ICPW) regression (He, 2018) to account for time-varying confounding at the pen level (Ji et al., 2020). Morbidity was abstracted from the records of antibiotic treatment due to BRD. Inverse conditional probability weighted logistic regression was used for *M. haemolytica* prevalence, mortality, and morbidity. Inverse conditional probability weighted linear regression was used for *M. haemolytica* Ct values. Inverse conditional probability weights consisted of stabilized weights that were estimated *via* conditional logistic regression (see Table 1 for a list of variables included in the estimation of propensity scores). Each outcome was regressed on *M. bovis* presence at previous time points, treatment group, and their interactions.

**Table 1:** Predictors used to estimate the propensity scores for *Mycoplasma bovis* prevalence at days 0, 7, and 14 post-arrival.

**Predictor**	**PS for Mb day 0**			**PS for Mb day 7**			**PS for Mb day 14**		
	**D0**	**D7**	**D14**	**D0**	**D7**	**D14**	**D0**	**D7**	**D14**
Mb				X			X	X	
Mh	X			X	X		X	X	X
Hs	X			X	X		X	X	X
Pm	X			X	X		X	X	X
BRSV	X			X	X		X	X	X
BW	X			X	X		X	X	X
Sex	X			X			X		
Ranch tag	X			X			X		
Morbidity					X			X	X

PS, propensity score; Mb, *Mycoplasma bovis* presence/absence; Mh, *Mannheimia haemolytica* presence/absence; Hs, *Histophilus somni* presence/absence; Pm, *Pasteurella multocida* presence/absence; BRSV, BRSV presence/absence; BW, body weight. Sex, whether the calf was a steer or bull. Ranch tag, presence/absence of a pre-existing ranch tag. D0-D14, days post-arrival in which the corresponding predictor was included in estimating the propensity scores.

The association of *M. bovis* presence on days 0, 7, and 14 post-arrival with time-to-first BRD antibiotic treatment was estimated using an ICPW Cox proportional hazards model using weights accounting for time-varying confounding and censoring. The administration of the first antibiotic treatment after arrival due to BRD was considered the event, and cattle that died or reached the end of the follow-up (70 days) without experiencing the event, were deemed to be right-censored. Since violations of the proportional hazard assumption were detected for *M. bovis* presence on days 0, 7, and 14 post-arrival using Schoenfeld residuals (Dohoo et al., 2012), those variables were included in an ICPW Cox model with time-varying coefficients. All statistical analyses were performed in Stata 17 (StataCorp, 2021), and the significance level was set to 0.05 *a priori*.

## Results

In this study, the vaccination groups did not differ in BW at arrival nor in the proportion of bulls and steers (Table 2). Twenty-six out of 270 cattle died before the end of the experiment (overall mortality: 9.6%). Therefore, 1,044 nasal swabs were used in the qPCR assays. The values of health parameters related to BRD at the end of the experiment are shown in Table 3. There was no significant difference in the proportion of cattle receiving antibiotics for BRD symptoms, or for retreatment, among the vaccination groups (Table 3). Likewise, the proportion of animals with chronic BRD and mortality did not significantly differ among the vaccination groups (Table 3). Of notice, we detected no significant differences between the cattle randomly selected for sampling and those not selected in the overall proportion of cattle receiving antibiotics for BRD (*P*-value: 0.846) or overall mortality (*P*-values: 0.32).

**Table 2:** Characteristics of the cattle after random allocation to the vaccination groups at arrival.

**Characteristics**	**Control (*****n** =* **90)**	**Intranasal (*****n** =* **90)**	**Parenteral (*****n** =* **90)**
Body weight at arrival	211.8 (19.2)	212.8 (17.2)	212.7 (17.8)
Steers	20 (0.22)	24 (0.27)	19 (0.21)
Bulls	70 (0.78)	66 (0.73)	71 (0.79)

Values between parentheses are proportions (for variables Steers and Bulls) or standard deviations (for variable body weight at arrival).

**Table 3:** Bovine respiratory disease-related health parameters at the end of the experiment.

**Health parameters**	**Control (*****n** =* **90)**	**Intranasal (*****n** =* **90)**	**Parenteral (*****n** =* **90)**
**Treatment for BRD**			
1	19 (0.21)	19 (0.21)	22 (0.24)
2	11 (0.12)	15 (0.17)	11 (0.12)
3	17 (0.19)	16 (0.18)	19 (0.21)
Chronic BRD	7 (0.08)	4 (0.04)	5 (0.06)
Mortality	10 (0.11)	8 (0.09)	8 (0.09)

Values between parentheses are proportions.

**Figure 1:**
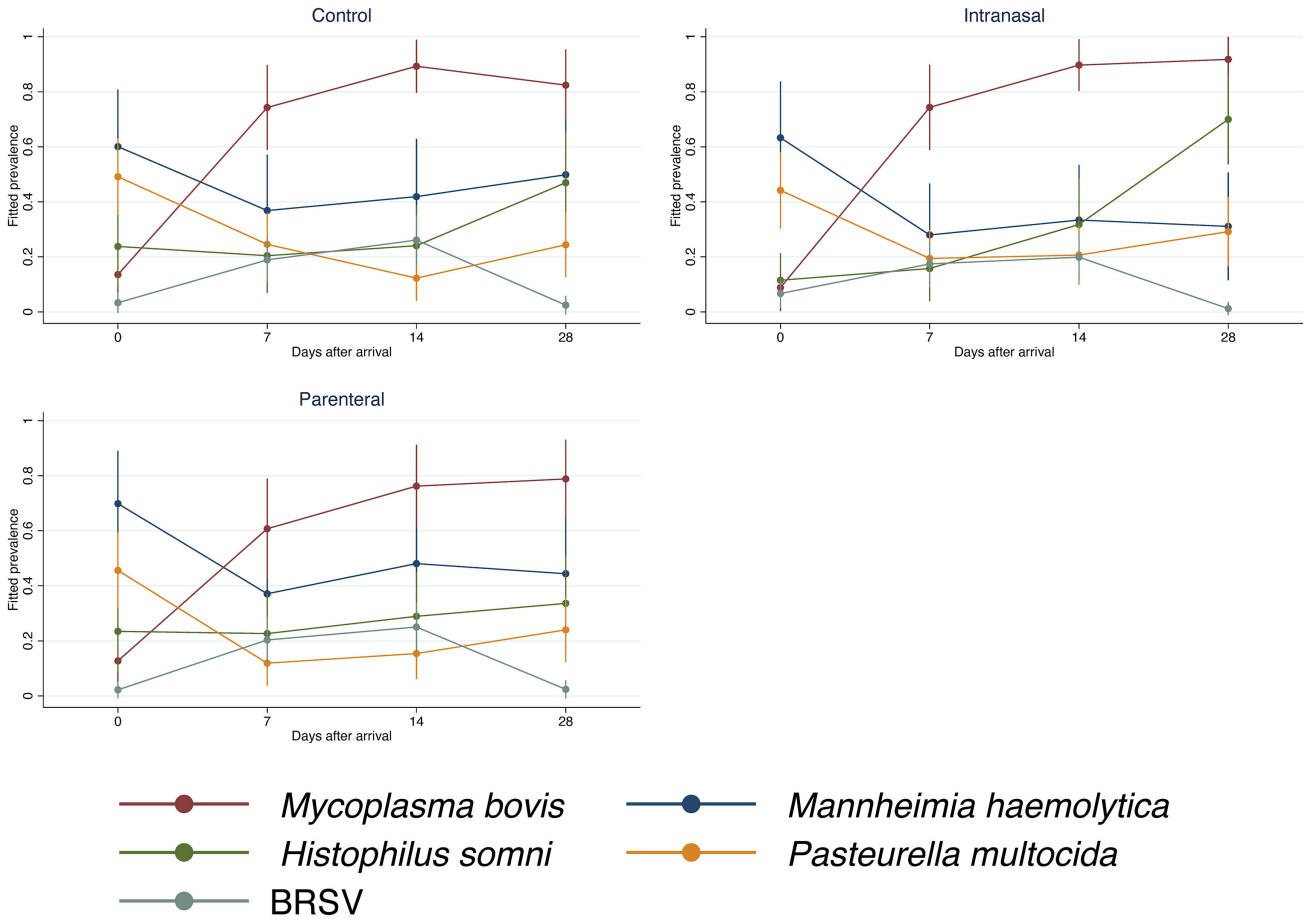
Prevalence of bovine respiratory disease pathogens by treatment. Control: unvaccinated negative control cattle. Intranasal: cattle vaccinated intranasally with a trivalent modified-live virus (MLV) respiratory vaccine (BHV-1, BRSV, and PI3V) with parenteral BVDV type I and type II vaccine. Parenteral: cattle vaccinated with a parenteral pentavalent MLV respiratory vaccine (BHV-1, BRSV, PI3V, BVDV type I and type II). The estimation of the prevalence values assumes that the animals share the same random effects.

The prevalence of all evaluated BRD pathogens changed significantly over time (overall test *P*-value < 0.0001). Highly heterogeneous longitudinal trends in the prevalence of BRD pathogens were observed throughout the study (Figure 1). For instance, in the control group, *M. bovis* prevalence increased from arrival until day 14 post-arrival and slightly decreased on day 28 post-arrival. By contrast, *M. haemolytica* showed a sharp decrease on day seven post-arrival with subsequent increments on the following 2 weeks.

On average, vaccination against BRSV significantly influenced the longitudinal trends of *M. bovis* and *H. somni* prevalence in the upper respiratory tract (overall joint test *P*-value = 0.0143 and 0.0001, respectively). A lower *M. bovis* prevalence was detected in the parenteral vaccination group when compared with the other two vaccination groups on day 7 (PD: −0.14, 95% Confidence Interval or 95% CI: −0.27 to −0.004, *P*-value: 0.043) and 14 post-arrival (parenteral vs. control PD: −0.13, 95% CI: −0.25 to −0.01, *P*-value: 0.029; parenteral vs. intranasal PD: −0.13, 95% CI: −0.25 to −0.02, *P*-value: 0.024). *Mycoplasma bovis* prevalence in the parenteral vaccination group continued to be lower than that in the intranasal vaccination group on day 28 post-arrival (PD: −0.13, 95% CI: −0.24 to −0.01, *P*-value: 0.027). The effect of the vaccination against BRSV on *H. somni* prevalence was only observed on day 28 post-arrival, in which *H. somni* prevalence in the upper respiratory tract of the intranasal vaccination group was significantly higher than the control (PD: 0.23, 95% CI: 0.07 to 0.39, *P*-value: 0.005) and parenteral vaccination group (PD: 0.36, 95% CI: 0.21 to 0.52, *P*-value < 0.001).

As measured by qPCR Ct values, the dynamics of *M. bovis* and *H. somni* also differed according to vaccination against BRSV (*P*-value = 0.0062 and < 0.0001, respectively). Significant differences were detected in *M. bovis* dynamics on days 7, 14, and 28 post-arrival, in which *M. bovis* qPCR Ct values in nasal swabs from cattle of the intranasal BRSV vaccine group were consistently lower (1.54 to 2.75 cycles, *P*-value < 0.05) than in cattle of the other two vaccination groups, except *M. bovis* qPCR Ct values of the control group on day seven and the parenteral vaccination group on day 28 post-arrival, which did not differ significantly from the corresponding *M. bovis* qPCR Ct values of the intranasal vaccination group. At day 28 post-arrival, *H. somni* qPCR Ct values were significantly lower in the intranasal vaccination group than in the other two vaccination groups (intranasal vs. control: −2.9 cycles, 95% CI: −4.23 to −1.62 cycles, *P*-value < 0.001; intranasal vs. parenteral: −3.52 cycles, 95% CI: −4.82 to −2.22 cycles, *P*-value < 0.001).

*Mycoplasma bovis* and *M. haemolytica* dynamics were positively associated in the upper respiratory tract of feedlot cattle. An increase in the qPCR Ct value of one cycle for *M. bovis* was associated, on average, with an increase in 0.08 cycles in the qPCR Ct value of *M. haemolytica* (95% CI: 0.02, 0.13) after adjusting for vaccination group and days post-arrival. *Mycoplasma bovis* was the only pathogen with a statistically significant association with *M. haemolytica* dynamics (Table 4).

**Table 4:** Association of *Mannheimia haemolytica* qPCR Ct values with those from *Mycoplasma bovis, Histophilus somni, Pasteurella multocida*, and BRSV.

**BRD pathogen**	**Mean difference in Mh qPCR Ct values**	**95% CI**	***P*** **-value**
Mb	0.08	(0.02, 0.13)	0.01
Hs	−0.02	(−0.09, 0.05)	0.536
Pm	0.01	(−0.06, 0.08)	0.739
BRSV	0.03	(−0.05, 0.12)	0.429

Mh, *Mannheimia haemolytica*; Mb, *Mycoplasma bovis* Ct value; Hs, *Histophilus somni* Ct value; Pm, *Pasteurella multocida* Ct value; BRSV, Bovine respiratory syncytial virus Ct value. 95% CI, 95% confidence interval. The mean difference in *Mannheimia haemolytica* Ct values was adjusted for the vaccination group and days post-arrival.

The presence of *M. bovis* in the upper respiratory tract of cattle on day seven post-arrival to the feedlot was associated with a significant increase in *M. haemolytica* prevalence on day 28 post-arrival, a significant decrease in *M. haemolytica* qPCR Ct values on day 28 post-arrival, and a significant increase in mortality, after adjusting for time-varying confounding (Table 5). Conversely, BRD morbidity on day 28 post-arrival was eight percentage points lower in cattle with *M. bovis* than cattle without *M. bovis* on arrival (Table 5). Additionally, *M. haemolytica* qPCR Ct values on day 28 post-arrival were on average higher in cattle with *M. bovis* than cattle without *M. bovis* on arrival (Table 5).

**Table 5:** Association of *Mannheimia haemolytica* prevalence and Ct values on day 28, mortality, and morbidity (on day 14 and 28) with the presence of *Mycoplasma bovis* on previous time points.

**Outcome**	**Mb day 0**	**Mb day 7**	**Mb day 14**
Mh prevalence day 28	n/e	0.45 (0.31, 0.6)*	0.11 (−0.12, 0.33)
Mh Ct values day 28	2.27 (0.46, 4.08)*	−2.89 (−4.2, −1.57)*	−1.2 (−2.92, 0.51)
Morbidity day 14	−0.05 (−0.23, 0.13)	0.1 (−0.02, 0.22)	n/a
Morbidity day 28	−0.08 (−0.14, −0.02)*	0.02 (−0.08, 0.11)	0.003 (−0.1, 0.1)
Mortality	−0.04 (−0.08, 0.003)	0.04 (0.002, 0.08)*	−0.007 (−0.1, 0.08)

Mh, *Mannheimia haemolytica*; Mb, *Mycoplasma bovis* presence/absence. Values are predictive margins (prevalence differences or mean differences accordingly) with 95% confidence intervals between parentheses. Asterisk (^*^) and boldfaced figures: *P*-value < 0.05; n/e, not estimable; n/a, not applicable.

During the study, 149 cattle received antibiotic treatment against BRD (the event in the survival analysis). Five cattle were right-censored because they died before receiving any antibiotic treatment against BRD. Additionally, 116 cattle had not experienced the event by the end of the experiment, and therefore, they were also right-censored. The data from 26 cattle could not be used in the survival analysis because the inverse conditional probability weights could not be computed.

**Figure 2:**
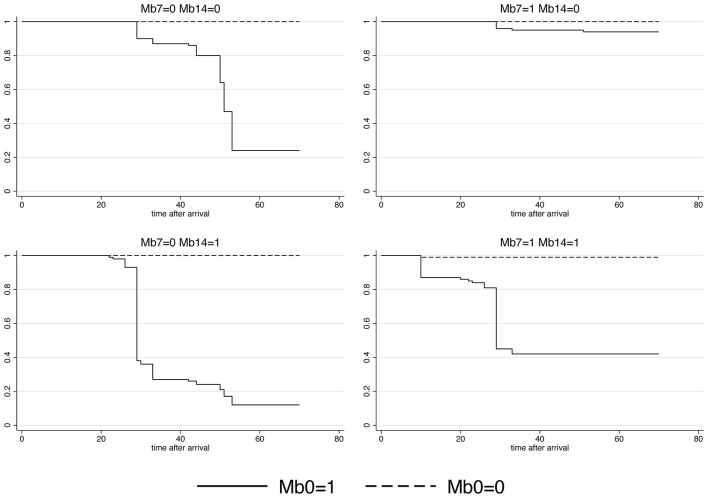
Survival curves for *Mycoplasma bovis* presence in the upper respiratory tract of cattle on days 0, 7, and 14 post-arrival to the feedlot. The event was the first antibiotic treatment against bovine respiratory disease. Mb0, *M. bovis* presence on arrival; Mb7, M. bovis presence on day seven post-arrival; Mb14, *M. bovis* presence on day 14 after arrival. The values 1 and 0 represent the presence and absence of *M. bovis*, respectively.

A highly heterogeneous association was observed between *M. bovis* presence and the time-to-first BRD antibiotic treatment. Significant time-varying coefficients for *M. bovis* presence were detected in all evaluated time points in the ICPW Cox model (*P* < 0.001). The shortest median time-to-first BRD antibiotic treatment was 29 days in cattle *M. bovis* positive on days 0, 7, 14 post-arrival and those positive on days 0 and 14. A median time-to-first BRD antibiotic treatment of 51 days was estimated for cattle *M. bovis* positive only on arrival. In the rest of the categories, the median time-to-first BRD antibiotic treatment was greater than 70 days (Figure 2).

## Discussion

This research showed that *M. bovis* and *M. haemolytica* are associated during the acute stages of BRD in feedlot cattle. This finding broadens our understanding of BRD pathogenesis and opens new avenues for the development of control strategies that do not rely in AMD. We observed that *M. bovis* prevalence increased in the upper respiratory tract of cattle 1 week after arrival to the feedlot, which was associated with a substantial increase in *M. haemolytica* prevalence 2 weeks later. Cattle consistently harboring *M. bovis* in the upper respiratory tract during the first 2 weeks at the feedlot were at a greater risk of receiving antibiotics as treatment for BRD. Additionally, we detected an effect of BRSV vaccination on *M. bovis* and *H. somni* dynamics in the upper respiratory tract, which demonstrates that interventions targeting one agent could result in unintended consequences in the respiratory microbiome.

*Mycoplasma bovis* is considered a commensal of the upper respiratory tract of cattle and has been documented to rapidly colonize cattle once they begin commingling in feedlots (Allen et al., 1991, 1992; Caswell and Archambault, 2008; Castillo-Alcala et al., 2012). We also observed a rapid spread of *M. bovis* among cattle after arrival at the feedlot. Since *Mycoplasma* are bacteria with an obligate parasitic lifestyle (Razin et al., 1998), *M. bovis* transmission in cattle should require close direct or indirect contact (Hermeyer et al., 2012b; Kanci et al., 2017; Haapala et al., 2018; Gille et al., 2020; Timonen et al., 2020). There is evidence that *M. bovis* can remain viable in materials commonly present in cattle production settings (Pfützner, 1984; Justice-Allen et al., 2010). However, the actual *M. bovis* persistence in the feedlot environment is unknown.

*Mannheimia haemolytica* is an upper respiratory tract commensal widely present in cattle (Rice et al., 2007; Confer, 2009; Klima et al., 2014). The observed decrease in *M. haemolytica* prevalence 1 week after arrival at the feedlot is very interesting. It may reflect changes in the location of the bacteria within the respiratory tract shortly after stressful events such as transportation to the feedlot (Grey and Thomson, 1971; Timsit et al., 2013; Caswell, 2014; Mosier, 2014; Boukahil and Czuprynski, 2015). Another possibility is the translocation of *M. haemolytica* to intracellular endosomes in bovine airway epithelial cells during the early stages of the infection (Cozens et al., 2019), which would reduce its detection in nasal swabs.

Traditionally, *M. bovis* has been associated with chronic stages of BRD. Furthermore, previous studies have documented that *M. haemolytica*-associated pneumonia during acute BRD predisposes cattle to subsequent infection by *M. bovis* in the lungs during chronic BRD, exhibiting caseonecrotic lesions (Adegboye et al., 1995; Rodriguez et al., 1996; Gagea et al., 2006a,b). However, our findings suggest that *M. bovis* presence may be a predisposing factor for an expansion of *M. haemolytica* in the upper respiratory tract of feedlot cattle within 1 month after arrival, in agreement with the findings of Gourlay and Houghton (1985), who documented that *M. bovis* increased the disease severity and extension of lesions in conventionally reared and gnotobiotic calves when inoculated before *M. haemolytica*. By contrast, experimental evidence indicates that *M. bovis* does not affect the pulmonary clearance of *M. haemolytica* (Lopez et al., 1982).

Harboring *M. bovis* in the upper respiratory tract during the first 2 weeks after arrival at the feedlot seems to be a risk factor for acute BRD. The observed increased hazard (and therefore risk) of receiving antibiotic therapy due to BRD symptoms was independent of the other BRD pathogens, suggesting that *M. bovis* may be participating in the pathogenesis of acute BRD, either directly or indirectly *via* changes in the respiratory microbiome. Timsit et al. (2018) observed that *M. bovis, M. haemolytica*, and *P. multocida* were enriched in the nasopharynx and trachea of cattle with bronchopneumonia.

We recognize that the scope of this work is not without limitations. When estimating associations between variables that change over time, time-varying confounding (Hernan and Robins, 2020) is a significant hurdle regardless of randomization at baseline. In this study, confounding due to variables at the pen level was accounted for entirely using conditional logistic regression models (Ji et al., 2020) to estimate the propensity scores for the presence of *M. bovis* during the first 2 weeks after arrival at the feedlot. Additionally, we incorporated within-pen variables to account for further confounding. However, bias due to confounding may still be present to a small degree (residual confounding) (Weiss and Koepsell, 2014). The Ct values without a standard curve or internal control to achieve absolute or relative quantification, respectively, include some degree of measurement error and therefore should be interpreted as an approximation. However, since the same amount of DNA per sample was used in the qPCR assays and lab personnel was blinded to treatment allocation, we consider that the measurement error in the Ct values is most likely non-differential. The antemortem diagnosis of BRD remains a clinical challenge. Therefore, non-differential misclassification of BRD could reduce the magnitude of the associations of interest (Rothman et al., 2008). Future research could expand upon the findings of this study by evaluating BRD dynamics in commercial feedlots, employing molecular techniques to track *M. bovis* and *M. haemolytica* at the strain level, and incorporating a more comprehensive approach to the diagnosis of BRD, adding thoracic ultrasound evaluations (Jourquin et al., 2022) for instance.

We are only beginning to grasp the subtleties of the interdependence among the bacterial and viral pathogens involved in BRD. Under the conditions of this study, we showed evidence suggesting that *M. bovis* could have a significant role during the acute phase of BRD, potentially favoring an imbalance in *M. haemolytica* abundance in the upper respiratory tract. Therefore, a reassessment of the relevance of *M. bovis* as an agent of BRD in cattle is warranted.

## Data availability statement

The raw data supporting the conclusions of this article will be made available by the authors, without undue reservation.

## Ethics statement

The animal study was reviewed and approved by West Texas A&M University Institutional Animal Care and Use Committee (IACUC # 2020.10.002).

## Author contributions

RV-C, SP, TM, PM, and JR: conceptualization, investigation, methodology, and writing-review and editing. RV-C and JR: funding acquisition and visualization. RV-C: formal analysis and writing-original draft preparation. PM and JR: supervision. All authors have read and agreed to the published version of the manuscript.

## Funding

This research was partially funded with RV-C start-up funds provided by Texas A&M University.

## Conflict of interest

The authors declare that the research was conducted in the absence of any commercial or financial relationships that could be construed as a potential conflict of interest.

## Publisher's note

All claims expressed in this article are solely those of the authors and do not necessarily represent those of their affiliated organizations, or those of the publisher, the editors and the reviewers. Any product that may be evaluated in this article, or claim that may be made by its manufacturer, is not guaranteed or endorsed by the publisher.
